# Immunomodulatory effect of mesenchymal stem cells in chemical-induced liver injury: a high-dimensional analysis

**DOI:** 10.1186/s13287-019-1379-6

**Published:** 2019-08-23

**Authors:** Jingqi Liu, Bing Feng, Yanping Xu, Jiaqi Zhu, Xudong Feng, Wenyi Chen, Xinyu Sheng, Xiaowei Shi, Qiaoling Pan, Jiong Yu, Xun Zeng, Hongcui Cao, Lanjuan Li

**Affiliations:** 1 State Key Laboratory for the Diagnosis and Treatment of Infectious Diseases, The First Affiliated Hospital, College of Medicine, Zhejiang University, 79 Qingchun Rd, Hangzhou City, 310003 China; 2 National Clinical Research Center for Infectious Diseases, The First Affiliated Hospital, College of Medicine, Zhejiang University, 79 Qingchun Rd, Hangzhou City, 310003 China; 3 College of Medicine, Zhejiang University, 866 Yuhangtang Rd, Hangzhou City, 310058 China; 4 Zhejiang Provincial Key Laboratory for Diagnosis and Treatment of Aging and Physic-chemical Injury Diseases, 79 Qingchun Rd, Hangzhou City, 310003 China

**Keywords:** Mass cytometry, Immune atlas, Mesenchymal stem cells, Immunomodulation, Adaptive immune cell, Innate immune cell

## Abstract

**Background:**

The efficacy of mesenchymal stem cell (MSC)-based therapy for acute liver injury (ALI) involves coordination with the hepatic immune system, a complex and coordinated network of immune-cell interactions. However, studies of the immunomodulatory effects of MSCs have focused on a limited number of cell subsets rather than a systematic assessment.

**Methods:**

Carbon tetrachloride (CCl_4_) was used to induce ALI in mice. To determine the efficacy of MSCs, ALI mice were injected with MSCs via the tail vein, and histopathological changes, survival rate, and the serum levels of liver enzymes were determined. To assess the immune response induced by MSCs, a mass cytometry panel of 43 metal isotope-tagged antibodies was used to characterize the hepatic immune compartment at days 1, 2, 3, and 7 after administration of MSCs or PBS.

**Results:**

MSC treatment significantly alleviated CCl_4_-induced ALI and improved the survival rate. MSC treatment also modulated the hepatic immune system in terms of the distribution of immune-cell subsets and the phenotype of single cells. During the injured phase, MSCs inhibited a systemic response by reducing the numbers of Ly6C^low^CD8^+^ T_RM_ cells, conventional NK cells, and IgM^+^IgD^+^ B cells; suppressing the activation of Ly6C^hi^CD8^+^ T_RM_ cells; downregulating MHC II and IgM expression in IgM^+^IgD^+^ B cells; and increasing the number of immunosuppressive monocyte-derived macrophages. During the recovery phase, MSCs promoted the retention of Ly6C^low^CD8^+^ T_RM_ cells and maintained the immunosuppressive activity of monocyte-derived macrophages. The response to MSC treatment differed between the injured and recovery phases, emphasizing the benefit of dynamic assessment of the immunomodulatory effects of MSCs.

**Conclusions:**

We determined the immunomodulatory effects of MSC treatment on the subtype distribution and phenotypes of hepatic immune cells.

**Electronic supplementary material:**

The online version of this article (10.1186/s13287-019-1379-6) contains supplementary material, which is available to authorized users.

## Background

Acute liver injury (ALI) induced by chemicals or viruses can progress rapidly to acute liver failure, for which liver transplantation is indicated [1]. However, because organ shortages and transplantation complications limit the applicability of this therapy, novel therapies are required. ALI involves the infiltration of immune cells, such as T cells, B cells, and natural killer (NK) cells [2, 3]. Immunosuppressive treatments and decreased inflammation reportedly promote repair after ALI [2]. Thus, transplantation of mesenchymal stem cells (MSCs) shows promise for ALI because of its immunomodulatory and immunosuppressive effects.

MSCs are multipotent stromal cells that can be isolated from various tissues and can differentiate into several lineages of cells, including osteoblasts, adipocytes, and chondrocytes [4]. Previous animal and clinical studies have shown that transplantation of MSCs ameliorates liver injury by inhibiting the activation and function of various immune cells, such as suppressing T cell proliferation, downregulating the proliferation and cytotoxicity of NK cells, and suppressing the maturation of monocytes into dendritic cells (DCs) [4–6]. Due to the limitations of flow cytometry, studies of the immunomodulatory effect of MSCs have focused on individual cell subsets. However, the immune system is a complex and mobile network of various cells and molecules, and therefore, systematic analyses of the interaction between the immune system and MSCs in ALI are needed.

High-dimensional mass cytometry combines flow cytometry and mass spectrometry. This technology uses stable heavy-metal isotopes in place of fluorophores and thus is not limited by spectral overlap of fluorophores and enables the simultaneous measurement of > 40 parameters at the single-cell level [7–10]. Therefore, mass cytometry enables systematic analyses of immune cell dynamics and system-wide assessment of the efficacy of MSCs against ALI.

In this study, we used high-dimensional mass cytometry to identify differences in the liver immune-cell landscape and dynamics in mice with carbon tetrachloride (CCl_4_)-induced ALI treated with MSCs or not. We profiled the immunomodulatory effects of MSCs on the hepatic immune system including the distribution and phenotypes of immune-cell subsets at single-cell resolution. Our data expand knowledge of the immunomodulatory effects of MSCs on the hepatic immune system, which will facilitate the development of MSC-based treatments for ALI.

## Methods

### MSC treatment of mice with ALI

**Fig. 1:**
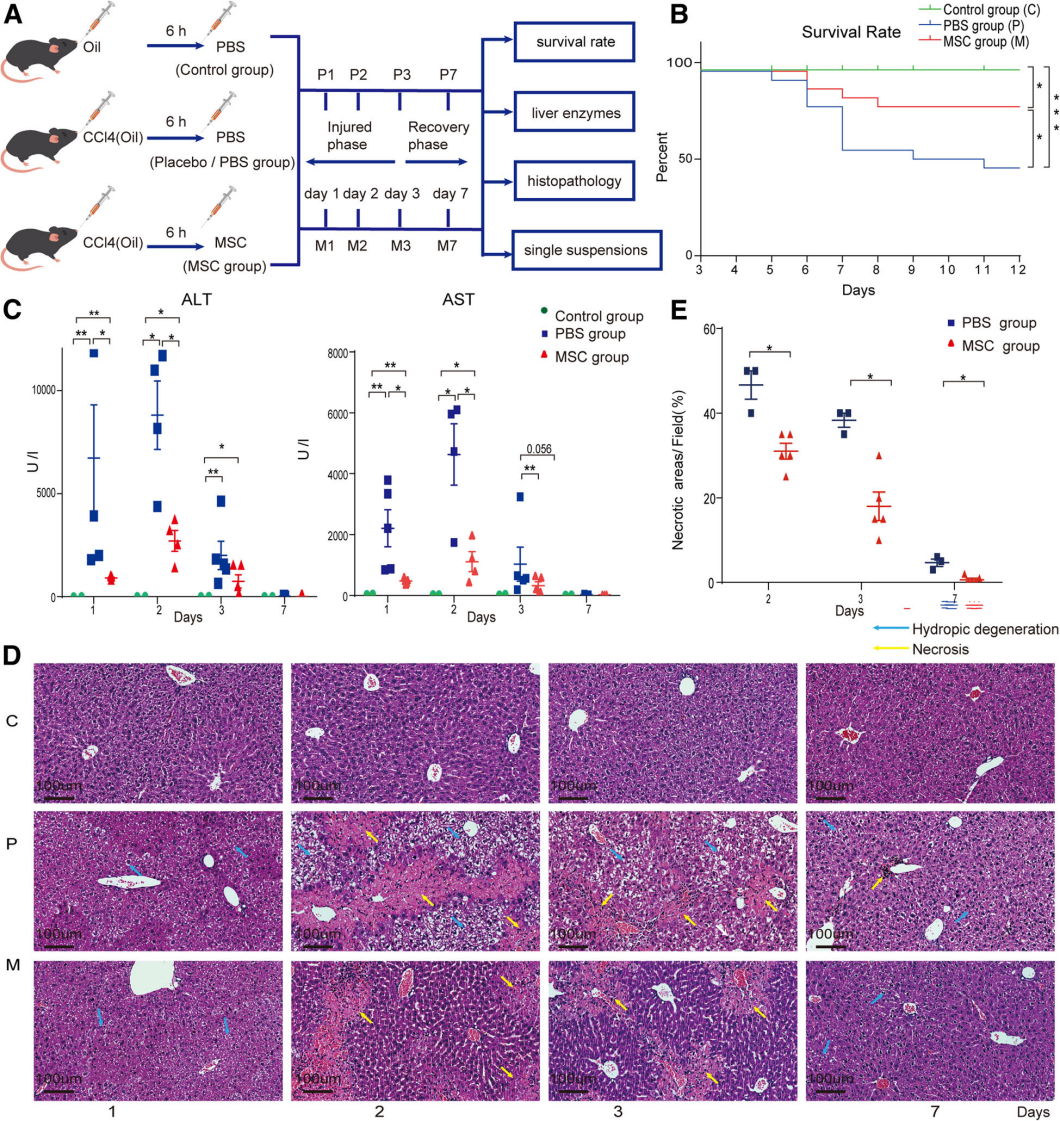
MSCs ameliorated CCl_4_-induced ALI. **a** Determination of the efficacy of MSCs in ALI. **b** Effect of MSC transplantation on the survival rate at 7 days (*n* = 22 per group). *P < 0.05 compared to the placebo (PBS) group by the Kaplan-Meier test. **c** Serum levels of ALT and AST at days 1, 2, 3, and 7 after transplantation; data are means ± SEM (*n* = 3–5 per group). *P < 0.05 compared to the placebo (PBS) group by the Mann-Whitney *U* test. **d** HE-stained images of the liver at days 1, 2, 3, and 7 after MSC (M) or PBS (P) administration. **e** Percentages of necrotic areas at days 2, 3, and 7 after transplantation. Data are means ± SEM (*n* = 3–5 per group). *P < 0.05 compared to the placebo (PBS) group by the Mann-Whitney *U* test

To determine the efficacy of MSCs against ALI, MSCs at passages 3 to 5 isolated from compact bone of C57BL/6 mice were characterized by inducing osteogenic and adipogenic differentiation and analyzing surface marker expression by flow cytometry (Additional file 1: Figure S1). Six- to eight-week-old male C57BL/6J mice were intraperitoneally administered 3 mL/kg of CCl_4_ (Sigma-Aldrich Co. LLC, St. Louis, MO, USA) diluted in olive oil (v/v, 50%, Sigma-Aldrich) to induce ALI. Control mice received olive oil alone. Six hours after CCl_4_ administration, the mice with ALI were divided into the MSC group (injected into the tail vein with 5 × 10^5^ MSCs in 0.1 mL of PBS containing 2% mice serum) and placebo (PBS) group (injected into the tail vein with an equal volume of PBS containing 2% mice serum). Control mice were injected with an equal volume of PBS containing 2% mice serum (control group). The mice were euthanized on days 1, 2, 3, and 7 after MSC or PBS administration, and liver tissue and serum were collected for histological and biochemical analyses. Mortality was recorded every 24 h until day 7 post-transplantation (Fig. 1a).

### Antibody labeling

A mass cytometry panel of 43 metal isotope-tagged antibodies (Additional file 1: Table S1) was used to evaluate the adaptive and innate immune cell populations in the mouse liver. The antibodies were conjugated to the indicated metal tags using a MaxPAR X8 Antibody Conjugation Kit (Fluidigm, San Francisco, USA) according to the manufacturer’s protocol. The conjugated antibodies were diluted to 200 mg/mL in Candor Antibody Stabilizer (Sigma) and titrated to the optimal concentrations.

### High-dimensional analysis

Single-liver-cell suspensions were washed once with 1 mL of fluorescence-activated cell sorting (FACS) buffer (PBS with 0.5% BSA and 0.02% NaN_3_) and incubated in 0.25 μM cisplatin (Fluidigm) for 5 min on ice to enable discrimination of dead cells. The cells were washed with FACS buffer, after which 20 mg/mL mouse/hamster/rat total IgG (Equitech-Bio, Inc., Cotton Gin Lane, Kerrville, USA) was added to block Fc receptors, and then, the cells were incubated on ice for 20 min. Next, the cells were reacted with an anti-CD49a-APC primary antibody (100 μL) for 30 min on ice. The single-cell suspensions were reacted with 100 μL of a metal-isotope-conjugated antibody cocktail (Additional file 1: Table S1) for 30 min on ice. The cells were washed twice and incubated in 0.03 μM Ir nucleic-acid intercalator (Fluidigm) in Fix and Perm Buffer (Fluidigm Sciences) at 4 °C overnight. The cells were washed with Perm Buffer (eBioscience Inc., San Diego, CA, USA) and stained with 100 μL of a metal isotope-conjugated intracellular antibody cocktail (Additional file 1: Table S1) in Perm buffer for 30 min on ice.

The cells were counted, resuspended at 0.6 × 10^6^/mL in distilled water containing 20% EQ. 4 beads (Fluidigm), and filtered through capFACS tubes (Corning). Mass cytometry data were acquired using a Helios system (Fluidigm Sciences) at ≤ 500 events per second.

Raw mass cytometry data in .fcs files were manually gated as live, singlet, and valid immune cells (Additional file 1: Figure S2). The data were subjected to the metal isotope beads normalization method. The gated cell populations were clustered using the X-shift algorithm in MATLAB. The signal intensities of the markers were transformed using Arcsinh with a cofactor of 5, and the transformed signal intensities were normalized as follows: the top 1% was excluded, and the maximum signal intensity of each marker was defined as the 99th percentile. All of the data were divided by this value, yielding single intensities for each channel of 0 to 1. Normalized marker expression levels were visualized as heatmaps. The percentage of each cluster was calculated as the percentage of live and singlet CD45^+^ cells. Clusters with proportions of less than 0.1% were ignored. To visualize the high-dimensional data in two dimensions, data from 10,000 randomly selected cells from each sample were processed using the nonlinear dimensionality reduction algorithm t-Distribution Stochastic Neighbor Embedding (t-SNE) [11–13]. viSNE maps were generated using MATLAB (MATLAB Release 2015b macOS 64-bit version, MathWorks, Inc., Natick, MA, USA).

### Statistical analyses

**Fig. 2:**
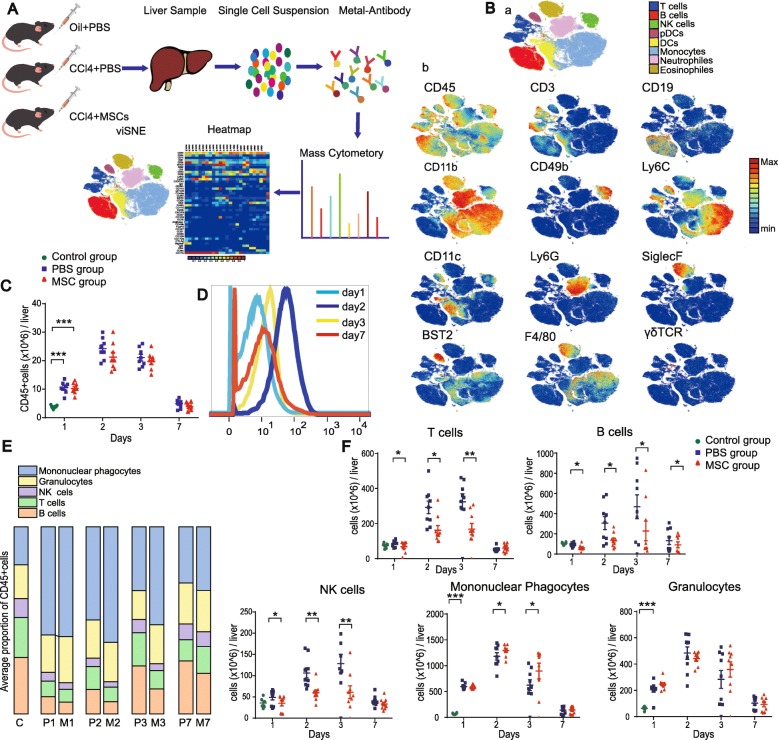
Identification of major immune-cell lineages in ALI. **A** Evaluation of the mouse hepatic immune system by mass cytometry. **B** (a) viSNE map of mouse hepatic immune-cell subsets. **B** (b) viSNE map colored according to the relative expression levels of markers. **C** Absolute numbers of CD45^+^ immune cells in the mouse liver (*n* = 6). **D** Overlay histograms of KI-67 expression in CD45^+^ immune cells in the injured liver over time. **E** Composition of CD45^+^ cells over time (*n* = 8–9). **F** Absolute numbers of T cells, B cells, NK cells, mononuclear phagocytes, and granulocytes over time (*n* = 8–9, *P < 0.05, **P < 0.01, and ***P < 0.001 by the paired *t* test)

The data are presented as means ± standard error of the mean (SEM). The significance of differences was assessed by the paired *t* test, the Kaplan-Meier test, and Pearson correlation coefficients using the Statistical Package for the Social Sciences (SPSS) (ver. 19.0; IBM Corp., NY, USA). The heatmap in Fig. 2 was generated in MATLAB; other heatmaps and three-dimensional bar plots were generated in Origin (ver. 2017SR2; OriginLab®, Northampton, MA, USA).

The procedures for animal experiments, isolation and culture of mouse MSCs, osteogenic and adipogenic differentiation assay, flow cytometry, biochemical tests, and preparation of total liver single-cell suspensions are described in the Additional file 1.

## Results

### MSCs ameliorate CCl_4_-induced ALI

To determine the efficacy of MSCs in ALI, we recorded the survival rate (Fig. 1a). At 7 days after MSC administration, the survival rate of the MSC group was significantly greater (*p* < 0.05) than that of the placebo group (82% vs. 54.5%) (Fig. 1b). Next, the serum levels of liver enzymes were determined (Fig. 1c and Additional file 1: Figure S3A). The alanine aminotransferase (ALT) and aspartate aminotransferase (AST) levels peaked at 2 days and then gradually recovered to an almost normal level at 7 days after MSC or placebo administration. The peak levels were significantly lower in the MSC group than in the placebo group (*p* < 0.01) (Fig. 1c). After PBS administration, sequential histopathological changes typical of ALI were observed microscopically, including diffuse hepatocyte hydropic degeneration on day 1 and diffuse hepatic necrosis on day 2; at day 7, only slight edema and individual necrotic cells were observed. In contrast, hepatic necrosis in the MSC group was markedly ameliorated on days 1, 2, and 3 after MSC administration compared to the placebo group and had recovered to almost normal at day 7 (Fig. 1d). The necrotic areas in the livers of MSC-treated mice showed remarkable recovery on days 2, 3, and 7 compared to the placebo group (Fig. 1e). Therefore, MSC administration significantly alleviated CCl_4_-induced ALI. Additionally, to exclude off-target effects, GFP^+^ MSCs were detected in suspensions of liver parenchymal cells from ALI mice (Additional file 1: Figure S3B-S3E), and no GFP^+^ MSC engraftment was detected by PCR in the lung, spleen, blood, lymph node, heart, or kidney at 1, 3, 5, or 7 days after MSC administration (not shown).

### High-dimensional mass cytometry analysis of the mouse liver immune system

To validate the mass cytometry results, we identified the major cell subsets (T cells, B cells, NK cells, and myeloid cells) by mass cytometry and fluorescence flow cytometry (Additional file 1: Figure S4A and S4B). The relative frequencies of each subset by mass cytometry were similar to those obtained by flow cytometry (Additional file 1: Figure S4C), confirming the reliability of the mass cytometry data. To comprehensively analyze the mouse liver immune landscape and the interaction between hepatic immune-cell subsets and MSCs, we characterized the liver immune compartment at days 1, 2, 3, and 7 after administration of MSCs or PBS (Fig. 2A).

### Identification of major immune-cell lineages in ALI

To generate a comprehensive view of the liver immune system, mass cytometry data were processed using the nonlinear dimensionality reduction algorithm t-SNE [11–13]. We analyzed the distribution of major immune cell lineages of mice livers in three groups (Fig. 2B–F, Additional file 1: Figure S5). A significantly larger number of immune cells were present in the livers of mice with ALI compared to control mice. The number of immune cells peaked at day 2 and gradually decreased up to day 7 (Fig. 2C). This pattern is consistent with the marked upregulation of Ki-67 expression on CD45^+^ liver immune cells after ALI, which peaked at day 2 and gradually decreased up to day 7 (Fig. 2D). These results, together with the serum levels of liver enzymes and histopathological changes, indicate that immune activation and liver injury are discernable by day 2 after ALI (the injured phase) and recover by day 7 (the recovery phase).

The composition and dynamics of the liver immune-cell populations differed among the three groups. In general, the adaptive lymphocyte subsets, B and T cells, were most abundant in the liver immune cell compartment of the control mice (30.1% and 21.3%, respectively); the mean proportion of mononuclear phagocytes was 20.3%. In mice with ALI, the proportion of mononuclear phagocytes dramatically increased (57.8%), whereas the proportions of B cells, T cells, and NK cells were markedly lower in the placebo than in the control group (Fig. 2E). Moreover, at 1–3 days after treatment, the numbers of T cells, B cells, and NK cells were significantly lower in the MSC-treated liver compared to the placebo-treated liver, whereas the number of mononuclear phagocytes was significantly greater in the MSC-treated liver. These results are consistent with previous reports that MSCs suppress the immune response by inhibiting the proliferation of B cells, T cells, and NK cells [6]. The numbers of T cells, NK cells, mononuclear phagocytes, and granulocytes did not differ significantly between the placebo and MSC groups at day 7 (*p >* 0.05), whereas the number of B cells was significantly lower in the MSC group at day 7 (Fig. 2F).

### Effects of MSC on the immune system during the injured and recovery phases

**Fig. 3:**
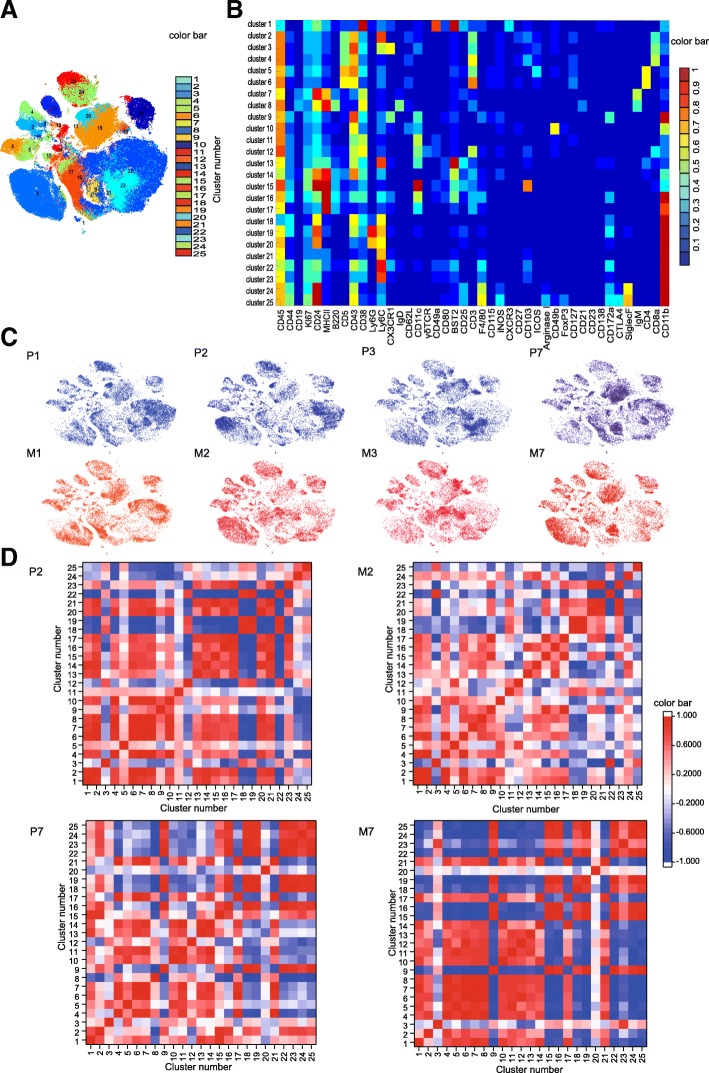
MSC-specific immune signatures in the injured and recovery phases. **a** viSNE map in Fig. 3b colored according to cluster. **b** Heatmap of the normalized expression of markers. **c** Liver immune-cell map generated using viSNE. **d** Heatmap of the Pearson coefficients of pairwise correlations of the interactions between liver immune-cell subsets

We subjected the mass cytometry data to an X-shift algorithm [11, 13], which partitioned the liver immune cells into distinct subsets in an unbiased manner (Fig. 2A). Up to 25 clusters were identified (Fig. 3a); the normalized expression of each cluster is shown as a heatmap (Fig. 3b). To globally visualize the distribution and dynamics of liver immune cells, their fingerprint-like characteristics were assessed using viSNE (Fig. 3c). This enabled the identification of clusters that differed between the placebo and MSC groups (Fig. 3c, Table 1, and Additional file 1: Table S2). The placebo and MSC-treated livers displayed distinct cellular signatures during the injured and recovery phases (Fig. 3c). To assess the immune response, we conducted pairwise correlation analyses of all liver immune-cell subsets (Fig. 3d). The marked differences between the placebo and MSC groups during the injured and recovery phases demonstrate that the MSC-induced immune coordination is dynamic and involves immune cell coordination and response. We thus evaluated the distribution and marker expression of clusters during the injured and recovery phases.

**Table 1:** Relationships between clusters and hepatic immune-cell populations

Subsets Label		Cluster	Subsets Label		Cluster
T cell	CD4^+^ T cell	5	Mononuclear phagocyte	DC	9
		6 (naïve CD4^+^T cell)			13
	CD8^+^ T cell	2 (Ly6C^hi^CD8^+^ T_RM_)			15
		3			16 (moDC)
		4 (Ly6C^low^CD8^+^ T_RM_)			17
	γδ T cell	12		Monocyte/macrophage	18
B cell		7 (IgM^+^ B cell)			21
		8 (IgM^+^IgD^+^ B cell)			22 (Monocyte-derived macrophage)
NK cell		1 (liver-resident NK cell)			23 (Monocyte)
		10 (conventional NK cell)	Granulocyte	Neutrophil	19
Unknown		11			20
		14		Eosinophil	24
					25

### Characteristics of MSC treatment-associated alterations in adaptive immune-cell subsets

**Fig. 4:**
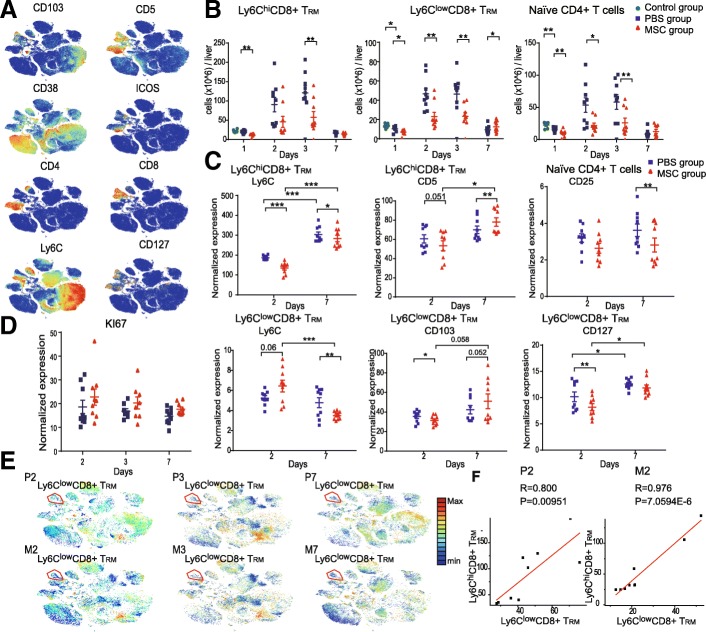
High-dimensional characteristics of MSC-specific alterations in T cell subsets. **a** viSNE maps of normalized expression levels of CD103, CD5, CD127, CD38, CD4, CD8, ICOS, and Ly6C. **b** Absolute numbers of Ly6C^low^CD8^+^ T_RM,_ Ly6C^hi^CD8^+^ T_RM_, and naïve CD4^+^ T cells over time (*n* = 8–9, *P < 0.05, **P < 0.01, and ***P < 0.001 by the paired *t* test). **c** Bar plots of normalized expression of cell-surface markers in the indicated cell subsets (*n* = 8–9, *P < 0.05, **P < 0.01, and ***P < 0.001 by the paired *t* test). **d** Bar plots of normalized KI67 expression. **e** viSNE map; colors indicate the KI67 expression levels. **f** Correlation plot of Ly6C^low^CD8^+^ T_RM_ and Ly6C^hi^CD8^+^ T_RM_ cells

X-shift and t-SNE analyses identified six T cell subsets, and paired mass cytometry analysis revealed their compositions and phenotypic alterations (Fig. 4 and Additional file 1: Figure S6A-S6C). Marker expression was similar in clusters 2 and 4, in which expression of CD3, CD8, CD5, and CD43 was high and that of CD127 was low. Cluster 4 expressed a high level of integrin CD103 and a low level of Ly6C and thus likely represents CD8^+^ tissue-resident memory T cells (Ly6C^low^CD8^+^ T_RM_) [14]. Cluster 2 expressed a low level of CD103 and a high level of Ly6C (Figs. 3a, b and 4a). Ly6C is associated with augmented effector function and the activation of T cells [15, 16]. We therefore inferred that cluster 2 (Ly6C^hi^CD8^+^ T_RM_) represents activated effector CD8^+^ T_RM_ cells.

During the injured phase, the number of Ly6C^low^CD8^+^ T_RM_ cells was markedly reduced, with lower expression of CD103 and higher expression of Ly6C in the MSC compared to the placebo group (Fig. 4b, c, and Additional file 1: Figure S6B). Additionally, Ly6C^low^CD8^+^ T_RM_ cells in the MSC group expressed a low level of CD127 (IL7Ra) (Fig. 4c and Additional file 1: Figure S6B). IL7 contributes to T_RM_ survival [17, 18]. Therefore, although there was no difference in KI67 expression by Ly6C^low^CD8^+^ T_RM_ cells between the MSC and placebo groups (Fig. 4d, e), the number of Ly6C^low^CD8^+^ T_RM_ cells was markedly reduced in the MSC group. Moreover, the number of Ly6C^hi^CD8^+^ T_RM_ cells was decreased and their expression of CD5 and Ly6C was lower in the MSC compared to the placebo group (Fig. 4b, c and Additional file 1: Figure S6B), suggesting that MSCs suppressed the activation of Ly6C^hi^CD8^+^ T_RM_ cells during the injured phase. Ly6C^hi^CD8^+^ T_RM_ cells were strongly positively correlated with Ly6C^low^CD8^+^ T_RM_ cells (Fig. 4f) in the MSC group. Cluster 6 was characterized by the absence of expression of CD38 and ICOS in CD4^+^ T cells (Figs. 3a, b and 4a) and thus represents naïve CD4^+^ T cells. The number of naïve CD4^+^ T cells was significantly reduced in the MSC group (Fig. 4b).

The number of Ly6C^low^CD8^+^ T_RM_ cells was markedly increased in the MSC group compared to the placebo group during the recovery phase (Fig. 4b), and their expression of Ly6C and CD103 (associated with the survival of CD8^+^ T_RM_ cells) was significantly lower and higher, respectively (Fig. 4c and Additional file 1: Figure S6C). This suggests that MSCs restored the phenotype of Ly6C^low^CD8^+^ T_RM_ cells and promoted their retention. Additionally, Ly6C^hi^CD8^+^ T_RM_ cells in the MSC group expressed a lower level of Ly6C and naïve CD4^+^ T cells showed a lower level of CD25 (IL-2Ra subunit) compared to the placebo group during the recovery phase. Therefore, MSCs inhibited the activation and effector function of Ly6C^hi^CD8^+^ T_RM_ cells and decreased the affinity of naïve CD4^+^ T cells for IL-2R.

**Fig. 5:**
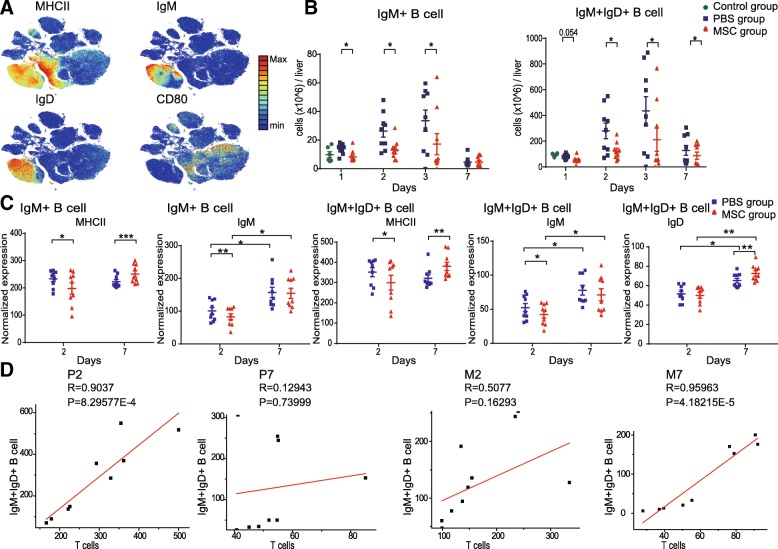
High-dimensional characteristics of MSC-specific alterations in B-cell subsets. **a** viSNE maps of normalized expression of MHC II, IgM, IgD, and CD80. **b** Absolute numbers of IgM^+^ and IgM^+^IgD^+^ B cells over time (*n* = 8–9, *P < 0.05, **P < 0.01, and ***P < 0.001 by the paired *t* test). **c** Bar plots of normalized expression of cell-surface markers in the indicated cell subsets (*n* = 8–9, *P < 0.05, **P < 0.01, and ***P < 0.001 by the paired *t* test). **d** Correlation plot of IgM^+^IgD^+^ B cells and T cells

We also analyzed the distribution and marker expression of B cells. The mouse liver B cell compartment contained two subsets: IgM^+^ B cells (cluster 7) and IgM^+^IgD^+^ B cells (cluster 8), both of which showed high expression of major histocompatibility complex class II (MHC II), but low expression of the costimulatory receptor CD80 (Figs. 3a, b, and 5a). IgM^+^IgD^+^ B cells were the most abundant subset in the B cell compartment. During the injured and recovery phases, the number of IgM^+^IgD^+^ B cells was lower with MSC treatment (Fig. 5b). The IgM^+^ B cells and IgM^+^IgD^+^ B cells showed lower expression of MHC II and IgM during the injured phase and higher expression of MHC II during the recovery phase in the MSC compared to the placebo group. IgM^+^IgD^+^ B cells showed higher expression of IgD in the MSC group during the recovery phase (Fig. 5c, Additional file 1: Figure S6D, S6E). Pelletier et al. reported that the expression of MHC II on B cells is associated with the regulation of T cell responses [19]; therefore, we analyzed the relationships between the B cell subsets and T cells. Interestingly, the numbers of IgM^+^IgD^+^ B cells and T cells were strongly positively correlated in the placebo group but not the MSC group during the injured phase. In contrast, during the recovery phase, the numbers of IgM^+^IgD^+^ B cells and T cells were strongly positively correlated in the MSC group but not in the placebo group (Fig. 5d). Collectively, these data indicate that the correlation between IgM^+^IgD^+^ B cells and T cells is associated with higher MHCII expression, consistent with the findings of Pelletier and colleagues.

Taken together, these results demonstrate the complex dynamics of the adaptive immune response induced by ALI, and that MSCs exerted distinct immunomodulatory effects during the injured and recovery phases.

### Characteristics of MSC-associated alterations in innate immune-cell subsets

**Fig. 6:**
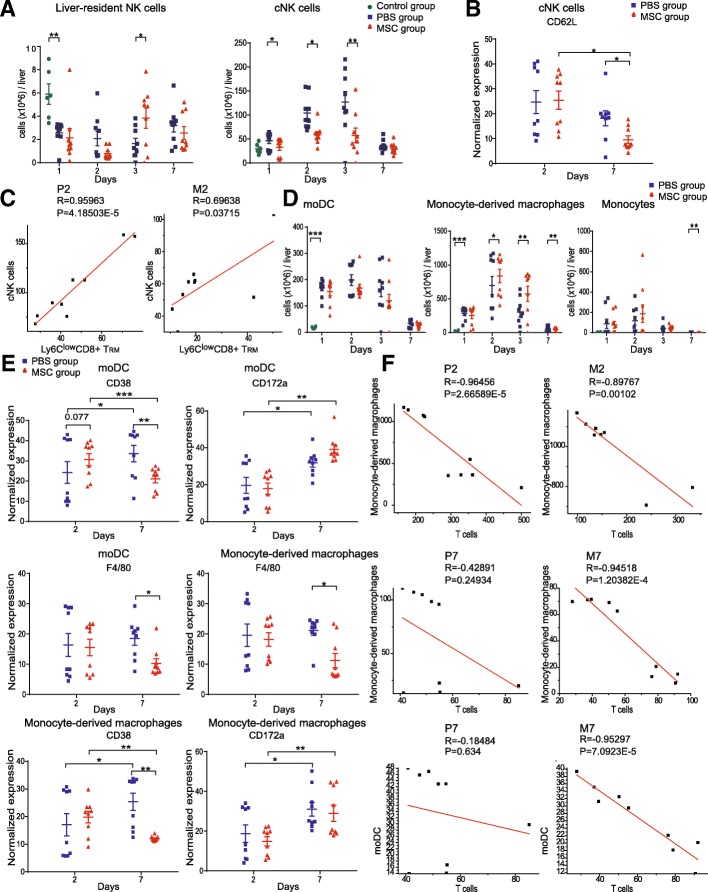
High-dimensional characteristics of MSC-specific alterations in innate-immune cell subsets. **a** Absolute numbers of liver-resident NK and cNK cells over time (*n* = 8–9, *P < 0.05, **P < 0.01, and ***P < 0.001 by the paired *t* test). **b** Bar plots of normalized expression of CD62L in cNK cells (*n* = 8–9, *P < 0.05, **P < 0.01, and ***P < 0.001 by the paired *t* test). **c** Correlation plot of cNK cells with Ly6C^low^CD8^+^ T_RM_. **d** Absolute numbers of moDC, monocyte-derived macrophages, and monocytes over time (*n* = 8–9, *P < 0.05, **P < 0.01, and ***P < 0.001 by the paired *t* test). **e** Bar plots of normalized expression of F4/80, CD38, and CD172a in moDC and monocyte-derived macrophages (*n* = 8–9, *P < 0.05, **P < 0.01, and ***P < 0.001 by the paired *t* test). **f** Correlation plot of moDC and monocyte-derived macrophages with T cells

We also analyzed the innate immune response in the different groups. In the mouse liver, NK cells were present as liver-resident NK cells (cluster 1, CD49a^+^CD49b^−^) and conventional NK (cNK) cells (cluster 10, CD49a^−^CD49b^+^) (Fig. 3a, b). cNK cells were the most abundant subset in the NK-cell compartment. During the injured phase, the number of cNK cells was decreased by MSC treatment (Fig. 6a). Moreover, MSC treatment decreased the expression of CD62L in cNKs during the recovery phase (Fig. 6b and Additional file 1: Figure S7A). The expression of CD62L on NK cells is correlated with a relatively mature NK cell phenotype and increased cytotoxicity [20]. Thus, MSCs suppressed the proliferation of cNK cells during the injured phase and decreased their cytotoxicity during the recovery phase. We also found that the number of cNK cells was strongly positively correlated with that of Ly6C^low^CD8^+^ T_RM_ cells in the MSC and placebo groups during the injured phase (Fig. 6c and Additional file 1: Figure S7B).

The myeloid cell compartment, which contains granulocytes and mononuclear phagocytes, presents antigens to prime naïve T cells and induces an adaptive immune response. Myeloid cells dominate the immune microenvironment of the injured liver and are vital for MSC-based treatment of ALI. Therefore, we mapped the distribution and dynamics of myeloid cell subsets.

X-shift and t-SNE analyses identified 13 myeloid-cell subsets, comprising five DC subsets (clusters 9, 13, 15–17), four monocyte subsets (clusters 18, 21–23), and four granulocyte subsets (clusters 19, 20, 24, 25) (Fig. 6d and Additional file 1: Figure S7C). Cluster 16 expressed CD11c, MHCII, Ly6C, and F4/80, consistent with monocyte-derived DCs (moDCs); CD80, CD44, KI67, BST2, and CX3CR1, indicative of activation; and a middling level of CD38 and the myeloid inhibitory immunoreceptor CD172a, also termed SIRPα or SHPS-1. Cluster 23 was Ly6C^hi^ monocytes, and cluster 22 expressed Ly6C, CD44, CX3CR1, F4/80, CD38, and CD172a (Fig. 3a, b and Additional file 1: Figure S5), suggesting that activation and differentiation to a macrophage-like state occurred. The number of moDCs (cluster 16) and monocyte-derived macrophages (cluster 22) decreased, and their expression of CD172a and CD38 increased in the placebo group, during the recovery phase compared to the injured phase (Fig. 6d, e, and Additional file 1: Figure S7D). CD38 is a marker of inflammation-induced differentiation of monocytes to DCs, is expressed by circulating monocytes and M1-polarized murine macrophages, and is a marker of the early innate immune response [21, 22]. Therefore, moDCs and monocyte-derived macrophages may be derived from circulating monocytes.

During the injured phase, the number of monocyte-derived macrophages was significantly increased by MSC treatment (Fig. 6d). In contrast, during the recovery phase, the number of monocyte-derived macrophages was markedly decreased by MSC treatment, and CD38 and F4/80 expression in monocyte-derived macrophages and moDCs was lower in the MSC than in the placebo group during the recovery phase (Fig. 6d, e, and Additional file 1: Figure S7E). Because DCs and monocytes present antigens to prime T cells, we evaluated their relationships. The numbers of moDCs, monocyte-derived macrophages, and T cells were strongly correlated. Also, the number of monocyte-derived macrophages was strongly negatively correlated with that of T cells in the MSC and placebo groups during the injured phase (Fig. 6f). In contrast, during the recovery phase, the numbers of moDCs and monocyte-derived macrophages were strongly negatively correlated with that of T cells in the MSC but not in the placebo group (Fig. 6f and Additional file 1: Figure S7F).

### Changes in CD43 after MSC treatment in the injured and recovery phases

The mass cytometry results suggested CD43, an abundant transmembrane glycoprotein, to be a marker of the immunomodulatory effects of MSCs in ALI. During the injured phase, CD43 expression in Ly6C^low^CD8^+^ T_RM_, Ly6C^hi^CD8^+^ T_RM_, and naïve CD4^+^ T cells and cNK cells was lower in the MSC than in the placebo group (Additional file 1: Figure S8A and S8C). During the recovery phase, CD43 expression in Ly6C^low^CD8^+^ T_RM_, Ly6C^hi^CD8^+^ T_RM_, and naïve CD4^+^T cells, cNK cells, and monocyte-derived macrophages was higher in the MSC group (Additional file 1: Figure S8B and S8C). However, the function of CD43 is elusive. CD43 has been reported to play both anti- and pro-adhesive roles and to suppress the activation and proliferation of T lymphocytes, NK cells, and monocytes [23]. However, other studies have found CD43 to promote the activation of lymphocytes [24–26]. The relevance of CD43 to the immunomodulatory effect of MSCs is unclear but is suggested by the similar changes in its expression in T cell subsets, NK cells, and monocyte-derived macrophages following MSC treatment.

## Discussion

The effective treatment of ALI is a concern worldwide, and MSC transplantation can ameliorate liver injury because of its immunomodulatory effects. There is an urgent need to understand the interactions between the complex hepatic immune system and MSCs in ALI. Here, we used mass cytometry to profile the MSC-induced alterations of the hepatic immune system in terms of both the distribution of immune-cell subsets and the phenotypes of single cells.

**Fig. 7:**
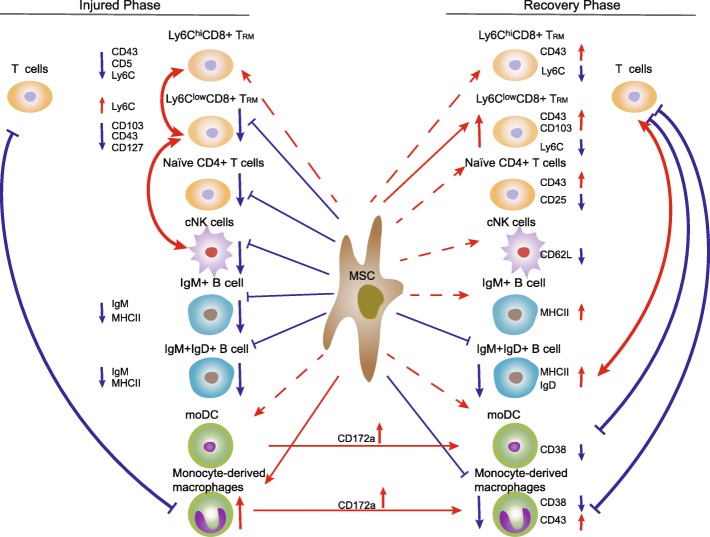
Immunomodulatory networks of MSCs in ALI

Our results showed that the MSC-induced immune response differed between the injured and recovery phases of ALI, including in terms of the composition and phenotype of immune cells as well as the relationships between immune-cell subsets (Fig. 7).

During the injured phase, MSC treatment decreased the number of Ly6C^low^CD8^+^ T_RM_ cells by downregulating the expression of CD127, the α-chain of the IL-7 receptor, which promotes the survival of T_RM_ cells [17]. Ly6C is associated with activated T cells, and Ly6C^hi^CD8^+^ T cells produce high levels of cytokines and granzymes to control acute infection [15, 16, 27]. MSCs promoted the activation of Ly6C^low^CD8^+^ T_RM_ cells by downregulating CD103 expression and upregulating Ly6C expression, but inhibited the activation of its effector counterpart, Ly6C^hi^CD8^+^ T_RM,_ by reducing the expression of CD5 and Ly6C. MSCs also reduced the number of B cells and suppressed their IgM production and antigen presentation by downregulating MHC II expression. The numbers of B cell subsets were strongly positively correlated with those of T cells in the placebo but not the MSC group, suggesting that MSCs downregulated MHC II on the surface of B cells to dampen interactions between T and B cells, thereby inhibiting the T cell response. Interestingly, CD8^+^ T_RM_ cells promote the recruitment of NK cells to injured tissues [28], and the number of cNK cells was strongly positively correlated with that of Ly6C^low^CD8^+^ T_RM_ cells in the MSC and placebo groups. Thus, the reduced number of Ly6C^low^CD8^+^ T_RM_ cells in the MSC-treated liver might have contributed to the paucity of cNK cells. MSC treatment increased the number of monocyte-derived macrophages, which was strongly negatively correlated with the number of T cells in the MSC and placebo groups, suggesting that monocyte-derived macrophages play an immunosuppressive role (Fig. 7). Collectively, these results suggest that MSCs inhibit the systemic immune response during the injured phase.

The MSC-induced systemic response was more complex during the recovery phase than the injured phase. The number and CD103 expression of Ly6C^low^CD8^+^ T_RM_ cells was markedly increased in the MSC group during the recovery phase [29]. This indicates that MSCs restored the phenotype of Ly6C^low^CD8^+^ T_RM_ cells, and upregulation of CD103 promoted the maintenance of Ly6C^low^CD8^+^ T_RM_ cells. Upon reinfection, hepatic T_RM_ cells are the first line of defense, quickly eliminating pathogens and promoting protective immunity in the liver [28]. Thus, the larger number of Ly6C^low^CD8^+^ T_RM_ cells in the MSC-treated liver may enhance protection against reinfection. Additionally, MSCs inhibited the effector function and activation of Ly6C^hi^CD8^+^ T_RM_ cells and decreased the CD25 (IL-2Ra subunit) expression of naïve CD4^+^ T cells (Fig. 7). Thus, IL-2 primes naïve T cells and promotes their Th1/Th2 differentiation by modulating the expression of IL-2R [30]. Hence, MSCs suppress the differentiation and priming of naïve CD4^+^ T cells.

MSCs reportedly either suppress B cell proliferation and downregulate costimulatory molecules (CD40, CD80, and CD86), or promote their expansion without affecting the expression of costimulatory molecules, secretion of antibodies, or antigen presentation [31, 32]. Therefore, the effects of MSCs on B cells may depend on the microenvironment. In this study, the number of IgM^+^IgD^+^ B cells was significantly reduced in the MSC-treated livers, and MSCs enhanced the antigen presentation and IgD secretion of the B cell subsets during the recovery phase (Fig. 7). The relevance of the composition and phenotype of IgM^+^IgD^+^ B cells to MSC-based therapy is unclear; further studies are needed.

During the recovery phase, MSCs downregulated CD62L expression in cNK cells. CD62L increases the cytotoxicity of NK cells [20], which may explain why MSCs suppressed the cytotoxic function of cNK cells. MSC treatment decreased the number of monocyte-derived macrophages, and the number of moDCs and monocyte-derived macrophages was strongly negatively correlated with that of T cells in the MSC but not in the placebo group (Fig. 7). CD172a suppresses the activation of myeloid cells [33], and CD38 enhances the engulfment of apoptotic cells and priming of the T cell response by myeloid cells [21]. We therefore hypothesized that the immunosuppressive role of moDCs and monocyte-derived macrophages in the MSC-treated liver is linked to the upregulation of CD172a and downregulation of CD38 during the recovery compared to the injured phase.

CD43 is important for the activation and proliferation of T lymphocytes, NK cells, and monocytes. CD43 reportedly inhibits the activation and proliferation of T lymphocytes and NK cells [23, 34] or promotes the activation and effects of lymphocytes [24–26]. In this study, CD43 expression in the T cell subsets, cNK cells, and monocyte-derived macrophages was downregulated during the injured phase and upregulated during the recovery phase. These results suggest that CD43 is a marker of the immunomodulatory effect of MSCs in ALI.

## Conclusions

We evaluated the effect of MSCs on the immune system of the injured liver and performed a system-wide assessment of their immunomodulatory effects. The systemic immune response to MSCs differed between the injured and recovery phases, including in terms of the distribution and phenotype of immune cells and the relationships between immune-cell subsets. Our data enhance understanding of the immunomodulatory effect of MSCs, which will facilitate the development of rational immunotherapies for ALI.

## Additional file

Additional file 1: **Table S1.** List of 43 metal isotope-tagged antibodies for mass cytometry. **Table S2.** Mean Proportions for all cell subsets of mouse liver immune cells by t-SNE/X-shift in our study. **Figure S1.** Characteristics of male C57BL/6 mouse MSCs at passage 3. **Figure S2.** Gating strategy for valid immune cells. **Figure. S3.** MSCs grafted into CCl_4_-induced injured livers and biochemical tests. **Figure S4.** Comparison mass cytometry and flow cytometry analysis of mouse liver four major cell subsets defined by traditional criteria. **Figure S5.** viSNE map of markers. **Figure S6.** High-dimensional characteristics of MSC-specific alterations in adaptive immune cell subsets. **Figure S7.** High-dimensional characteristics of MSC-specific alterations in innate immune cell subsets. **Figure S8.** Changes in CD43 after MSC treatment in the injured and recovery phases. (DOC 10303 kb)

## Data Availability

All data generated or analyzed during this study are included in this article.

## Abbreviations

ALI: Acute liver injury
ALT: Alanine aminotransferase
AST: Aspartate aminotransferase
CCl_4_: Carbon tetrachloride
cNK cell: Conventional natural killer cell
DC: Dendritic cell
GFP: Green fluorescent protein
moDC: Monocyte-derived dendritic cell
MSC: Mesenchymal stem cell
NK cell: Natural killer cell
