# Supplementary material for: Immunomodulatory effect of mesenchymal stem cells in chemical-induced liver injury: a high-dimensional analysis
Source: Stem Cell Res Ther. 2019 Aug 23;10:262. doi: 10.1186/s13287-019-1379-6 (PMC6708172; doi:10.1186/s13287-019-1379-6)

**SUPPLEMENTARY DATA**

**Immunomodulatory effect of mesenchymal stem cells in chemical-induced liver injury: A high-dimensional analysis**

Jingqi Liu^1^, Bing Feng^1^, Yanping Xu^1^, Jiaqi Zhu^1^, Xudong Feng^1^, Wenyi Chen^1^, Xinyu Sheng^1^, Xiaowei Shi^3^, Qiaoling Pan^1,2^, Jiong Yu^1,2^, Xun Zeng^1,2^, Hongcui Cao†^1,2, 4^, Lanjuan Li^1,2^

1 State Key Laboratory for the Diagnosis and Treatment of Infectious Diseases, the First Affiliated Hospital, College of Medicine, Zhejiang University, 79 Qingchun Rd., Hangzhou City 310003, China

2 National Clinical Research Center for Infectious Diseases, The First Affiliated Hospital, College of Medicine, Zhejiang University, 79 Qingchun Rd., Hangzhou City 310003, China

3 College of Life Science, Zhejiang University, 866 Yuhangtang Road, Hangzhou 310058, China

4 Zhejiang Provincial Key Laboratory for Diagnosis and Treatment of Aging and Physic-chemical Injury Diseases, 79 Qingchun Rd., Hangzhou City 310003, China

**†Corresponding author:**

Hongcui Cao, M.D.

State Key Laboratory for the Diagnosis and Treatment of Infectious Diseases, the First Affiliated Hospital, College of Medicine, Zhejiang University; National Clinical Research Center for Infectious Diseases, The First Affiliated Hospital, College of Medicine, Zhejiang University, 79 Qingchun Rd., Hangzhou City 310003, China. Tel: 86-571-87236451; Fax: 86-571-87236459

E-mail: [hccao@zju.edu.cn](mailto:hccao@zju.edu.cn)

**SUPPLEMENTARY METHODS**

**Animals**

Wild-type and enhanced green fluorescent protein (GFP)–transgenic C57B/L6 mice were purchased from Shanghai SLAC Laboratory Animal Co., Ltd. and Nanjing Biomedical Research Institute of Nanjing University, respectively. Female mice were backcrossed to C57BL6 male mice to produce younger male C57/BL6 mice for isolation of MSCs. Animals were allowed access to food and water *ad libitum* and housed under specific-pathogen-free conditions in the animal facility under a controlled light and dark cycle. All animal experimental procedures were conducted according to a protocol approved by the Ethics Committee of the First Affiliated Hospital of Zhejiang University.

**Isolation and culture of mouse MSCs**

We isolated and cultured MSCs as described by Zhu *et al* . Two- to three-week-old wild-type or GFP–transgenic C57BL/6 male mice were euthanized by cervical dislocation. Humeri, tibiae, and femurs were disconnected from the trunk, the soft tissue was cleaned, the epiphyses were removed, and marrow was flushed out thoroughly with 3 mL α-minimal essential medium (α-MEM, HyClone^TM^ GE Healthcare Life Sciences, Logan, USA) until the bones became pale. The compact bones were chopped into approximately 1–2 mm^3^ pieces, transferred to 15 mL tubes (Corning Inc., Corning, NY, USA) containing 2 mg/mL collagenase II digestion solution (Gibco® by Life Technologies, Grand Island, USA), and incubated at 37°C for 1.5 h with continuous rotation. The enzyme-treated bone chips were suspended in 7.5 mL C57BL/6-MSC special complete MEM (OriCell™ C57BL/6 MSC complete Medium, Cyagen Biosciences, Guangzhou, China) and incubated at 37°C in a 5% CO_2_ incubator (HERAcell®150, Thermo Fisher Scientific Inc.). At day 3, to remove non-adherent cells the complete MEM was replaced. At day 5 after isolation, adherent MSCs were harvested using 0.25% trypsin–EDTA (Invitrogen, Carlsbad, CA, USA) and resuspended in fresh complete MEM; complete MEM was replaced every 48 h. MSCs at passage 3 were considered purified and used in subsequent experiments.

**Osteogenic and adipogenic differentiation assay**

To induce osteogenic differentiation, MSCs were cultured in osteoinductive medium (OriCell™ C57BL6 MSC Osteogenic Differentiation Medium, Cyagen Biosciences) for 4 weeks with the appropriate supplements, followed by Alizarin Red S (Cyagen Biosciences) staining for osteocytes.

To induce adipogenic differentiation, MSCs were maintained in adipogenic culture medium (OriCell™ C57BL6 MSC Adipogenic Differentiation Medium, Cyagen Biosciences) with the appropriate supplements. After 4 weeks of adipogenic induction, the cultures were stained with Oil red O (Cyagen Biosciences).

**Flow cytometry analysis**

To identified the mouse MSC, cultured MSCs were harvested and incubated with anti-PE-CD44 (IM7; BioLegend®, Enabling Legendary Discovery®, San Diego, USA), PE-SCA-1 (D7; BioLegend), APC-CD29 (HMβ1–1; BioLegend), PECy7-CD31 (390; BioLegend), PerCP-CD86 (GL-1; BioLegend), APCCy7-CD11b (M1/70; BioLegend), PECy7-CD45 (30-F11; BioLegend), and APCCy7-MCH II-1A (M5/114.15.2; BioLegend) antibodies and corresponding isotype controls (Biolegend) for 30 min at 4°C in the dark. The MSCs were washed twice with phosphate-buffered saline (PBS, Hangzhou Gino Bio-pharmaceutical Technology Co. Ltd., Zhejiang, China) containing 0.5% bovine serum albumin (BSA, Sangon Biotech Corp., Shanghai, China), and analyzed by flow cytometry (BeamCyte-1026, Beamdiag, Changzhou, China).

To validate the mass cytometry results using fluorescence flow cytometry by traditional manual gating strategies, the single-cell suspensions of mice hepatic purified nonparenchymal cells were incubated with a mixture of fluorochrome-conjugated antibodies specific to mouse: FITC-CD45 (30-F11; Becton Dickinson (BD), San Jose, USA), BV786-CD3 (45-2C11; BD), PE-CD19 (6D5; BioLegend), APC-CD11b (M1/70; BD), BV421-CD49b (DX5; BD) and corresponding isotype controls (were purchased either from BD or Biolegend) for 30min at 4°C in the dark, then multiparameter analyses were done on Fortessa instrument (BD) and analyzed by FlowJo (Tree Star, Ashland, USA).

**Biochemical tests**

Liver enzyme levels were measured using a dry chemistry analyzer (DRI-CHEM 4000ie; Fujifilm Corp., Tokyo, Japan) according to the manufacturer’s instructions.

**Histological analyses**

Liver specimens were fixed in 4% paraformaldehyde and embedded in paraffin. Sections (5 µm) were stained with hematoxylin and eosin according to a standard methodology. Necrotic areas were analyzed using a Nanozoomer 2.0-RS scanner (Hamamatsu, Japan).

**Preparation of total liver single-cell suspensions**

Liver nonparenchymal cell isolation: Mice were anesthetized and the livers were perfused with 10 mL PBS for 2 min at 37°C. Gallbladders were removed and the livers were cut into small fragments, digested using a Mouse Liver Dissociation Kit (Miltenyi Biotec, Bergisch Gladbach, Germany), and homogenized using a gentleMACS C tube and a GentleMACS™ Dissociator (Miltenyi Biotec) according to the manufacturer’s instructions. The homogenate was filtered through a 100 µm cell strainer (Falcon®, Corning Incorporated, NY, USA), washed with 5 mL DMEM supplemented with stable glutamine to remove large particles, and centrifuged for 10 min at 300 × g. Single cells were recovered by suspension in 36% Percoll (GE Healthcare, Uppsala, Sweden) and centrifugation for 5 min at 600 × g. Finally, to lyse red blood cells (RBCs), cell pellets were resuspended in 3 mL AKC lysis buffer (Gibco) and incubated for 5 min at room temperature. The cell suspensions were washed and resuspended in FACS buffer (0.5% BSA, 0.02% sodium azide).

Liver parenchymal isolation: The liver parenchymal cell isolation procedure is based on a slightly modified two-step collagenase perfusion method described by Papeleu et al and Hirata et al . Mice were anesthetized and the livers were perfused with dispase (Gibco) and collagense IV (Gibco) solution, the livers were excised and filtered through a 100 µm cell strainer (Falcon®, Corning Incorporated, NY, USA). Freshly isolated liver parenchymal cell suspensions were centrifuged twice for 75 seconds at 50 × g. Then purified liver parenchymal cell were harvested.

**Supplementary refereneces**

1. Zhu H, Guo ZK, Jiang XX, Li H, Wang XY, Yao HY, et al. 11 A protocol for isolation and culture of mesenchymal stem cells from mouse compact bone. Nat Protoc. 2010; 5: 550-60.

2. Papeleu P, Vanhaecke T, Henkens T, Elaut G, Vinken M, Snykers S, et al. Isolation of rat hepatocytes. Methods Mol Biol. 2006; 320: 229-37.

3. Hirata M, Amano K, Miyashita A, Yasunaga M, Nakanishi T, Sato K. Establishment and characterization of hepatic stem-like cell lines from normal adult rat liver. J Biochem. 2009; 145: 51-8.

**Supplementary tables**

**Table S1: List of 43 metal isotope-tagged antibodies for mass cytometry**

**Table S2: Mean proportions for all cell subsets of mouse liver immune cells by t-SNE/X-shift in our study**

**Supplementary figures and figure legends**

**Figure S1****. Characteristics of male C57BL/6 mouse MSCs at passage 3**. **(A):** C57BL/6 MSCs are fibroblast-like cells at passage 3 (left); **(B)** and **(C):** Differentiation of MSCs into (middle) osteocytes (×10) and (right) adipocytes (×20). **(D):** Flow cytometry of cell-surface markers: MSCs expressed CD29, CD44, and Sca-1, but not CD31, CD86, CD45, CD11b, or MHC I-1a.


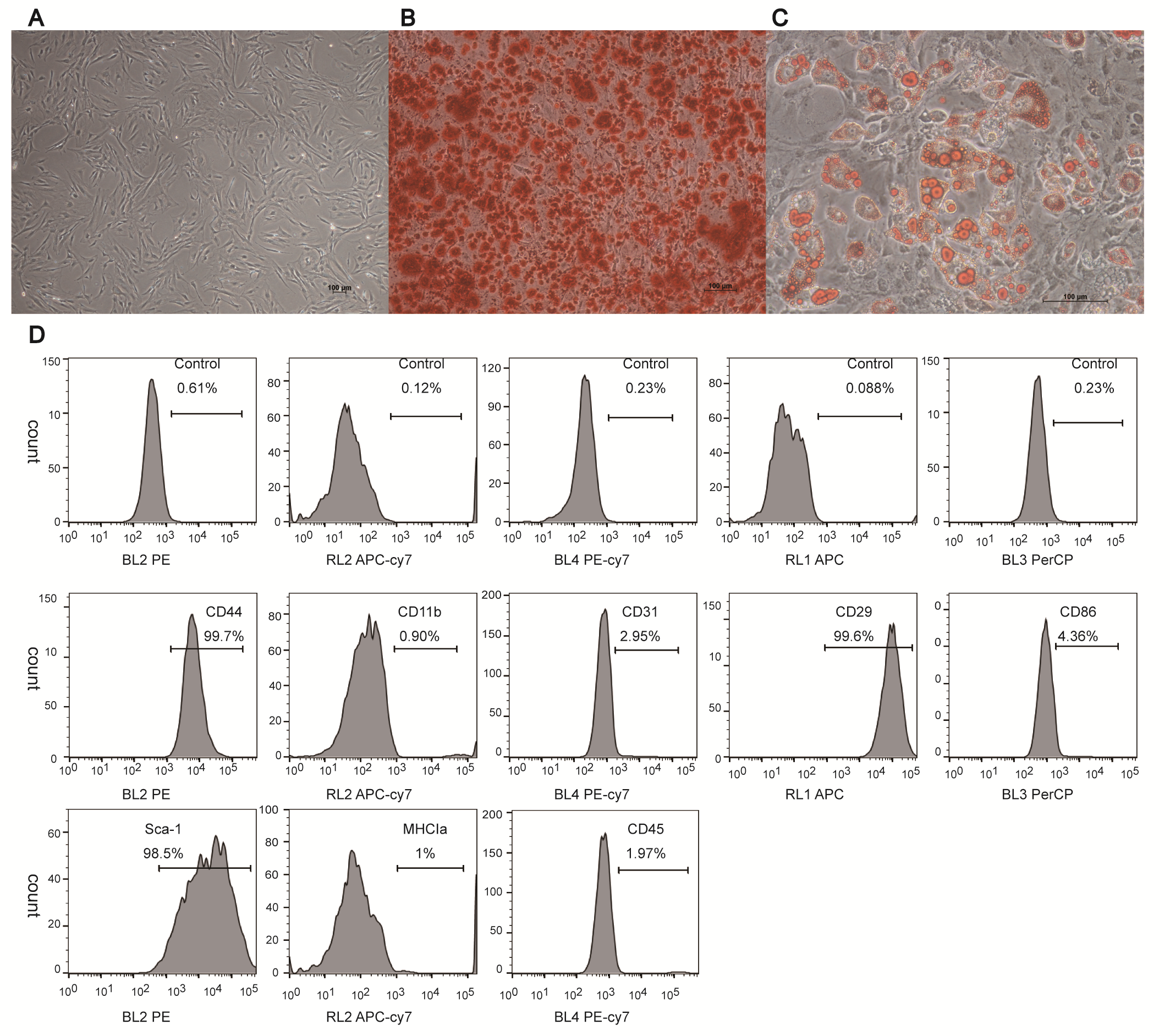


**Figure S2**. **Gating strategy for valid immune cells:** Cells were gated as cells (left), singlet and live cells (middle), and CD45^+^ cells (right).


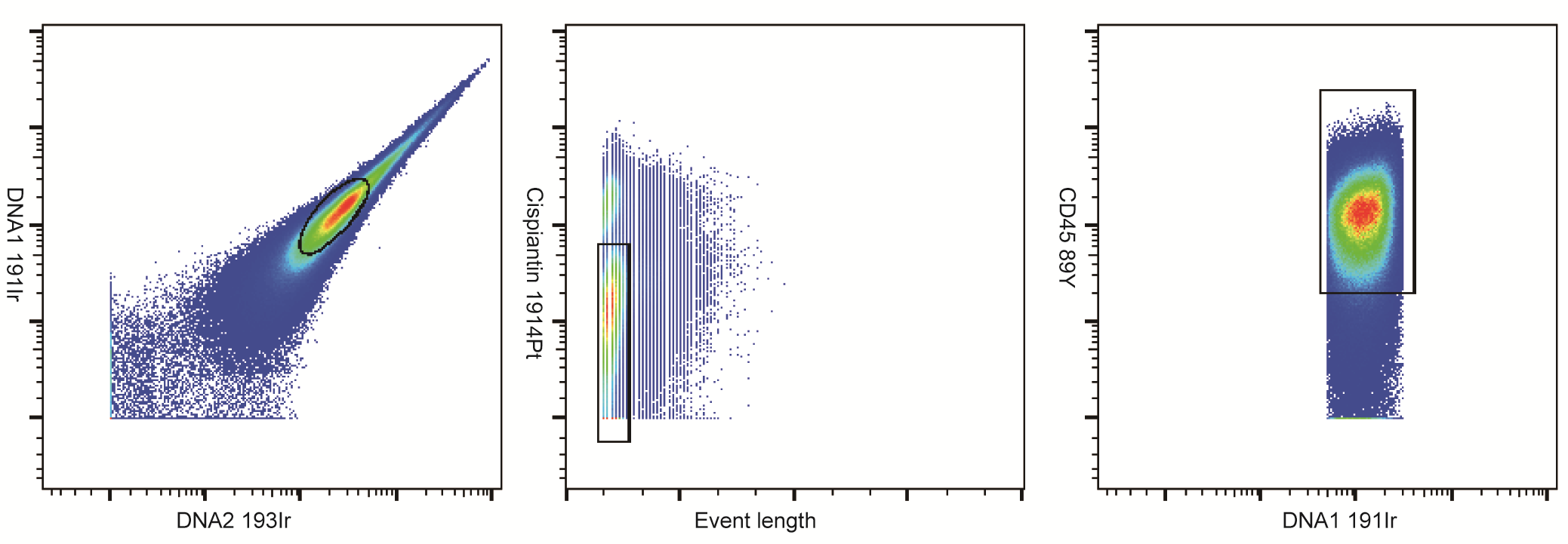


**Figure** **S3. MSCs grafted into CCl_4_-induced injured livers and biochemical tests. (A):** Mean ± SEM serum levels of CHE, GGT, ALP, and TBIL at days 1, 2, 3, and 7 after transplantation (n = 3–5 per group, by the Mann-Whitney U-test). **(B):** Images show GFP^+^ MSCs (green) under confocal microscopy, GFP, enhanced green fluorescent protein; **(C):** Images show liver parenchymal cell smear of placebo-treated groups under confocal microscopy. **(D):** Images show liver parenchymal cell smear of MSC treated groups under confocal microscopy (GFP^+^ MSCs were green). **(E):** The numbers of grafted GFP^+^-MSCs per field in MSC group at day 1, 3, 5, 7 after MSCs administration (n=20).


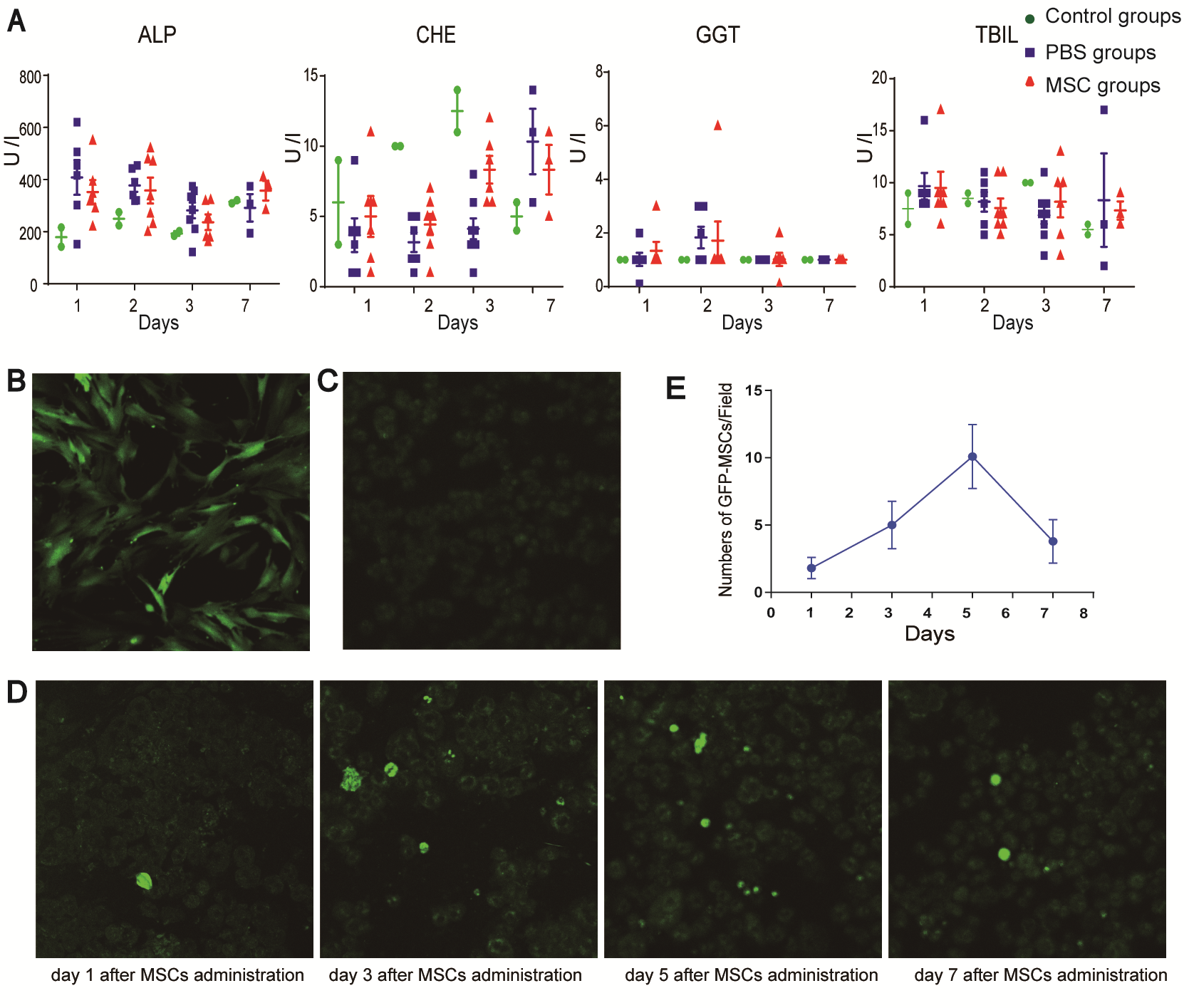


**Figure S4** **Comparison mass cytometry and flow cytometry analysis of mouse liver four major cell subsets defined by traditional criteria.** **(A):** Mass cytometry analysis of liver immune cell subsets defined by manual gating strategy for a representative mouse; **(B):** Flow cytometry analysis of liver immune cell subsets defined by manual gating strategy for a representative mouse; **(C):** Mean frequency of four major cell subsets obtained by mass cytometry and flow cytometry through manual gating strategy (n =6, **P <*0.05, ***P <*0.01, and ****P <*0.001 by the paired *t*-test).


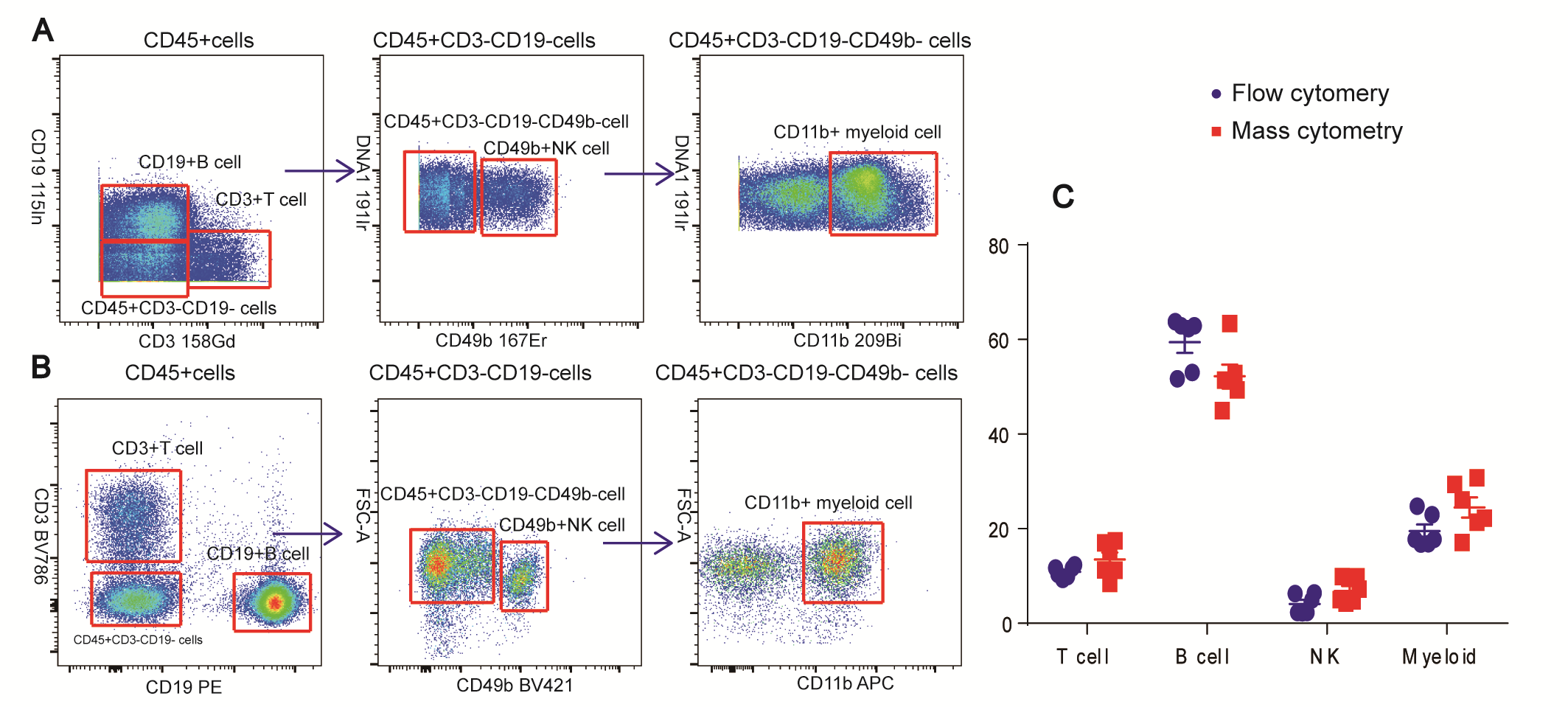


**Figure** **S5. viSNE map of markers.** Colors indicate relative expression levels.


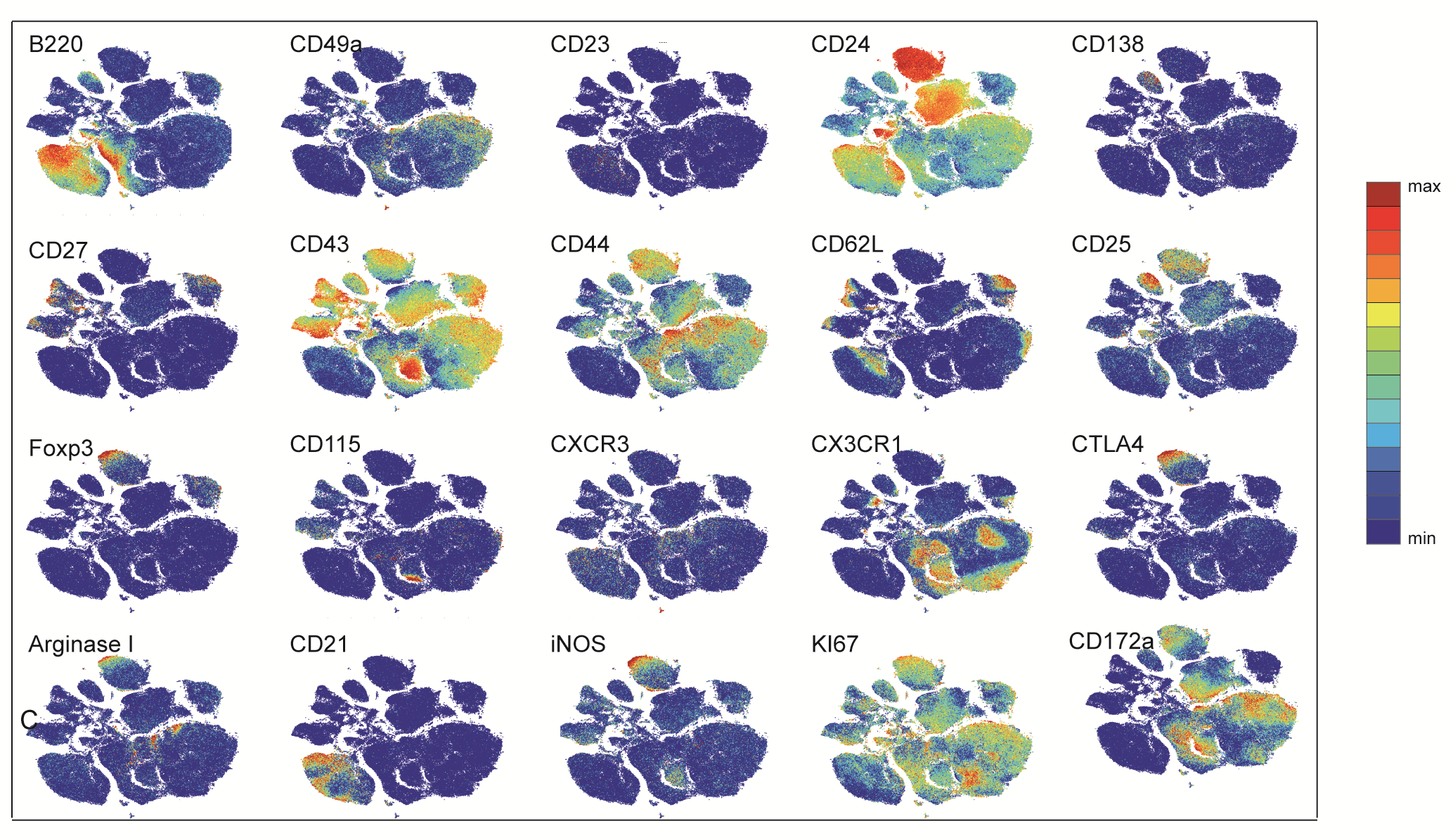


**Figure S6.** **High-dimensional characteristics of MSC-specific alterations in adaptive immune cell subsets.** **(A):** Absolute numbers of cluster 3, cluster 5 and cluster 12 over time (n = 8–9, **P <*0.05, ***P <*0.01, and ****P <*0.001 by the paired *t*-test). **(B**-**E)**: Representative overlay histograms of the expression of cell-surface markers on Ly6C^hi^CD8^+^ T_RM_, Ly6C^low^CD8^+^ T_RM_, naïve CD4^+^T cells IgM^+^ B cell and IgM^+^IgD^+^ B cell in the MSC and placebo groups.

**
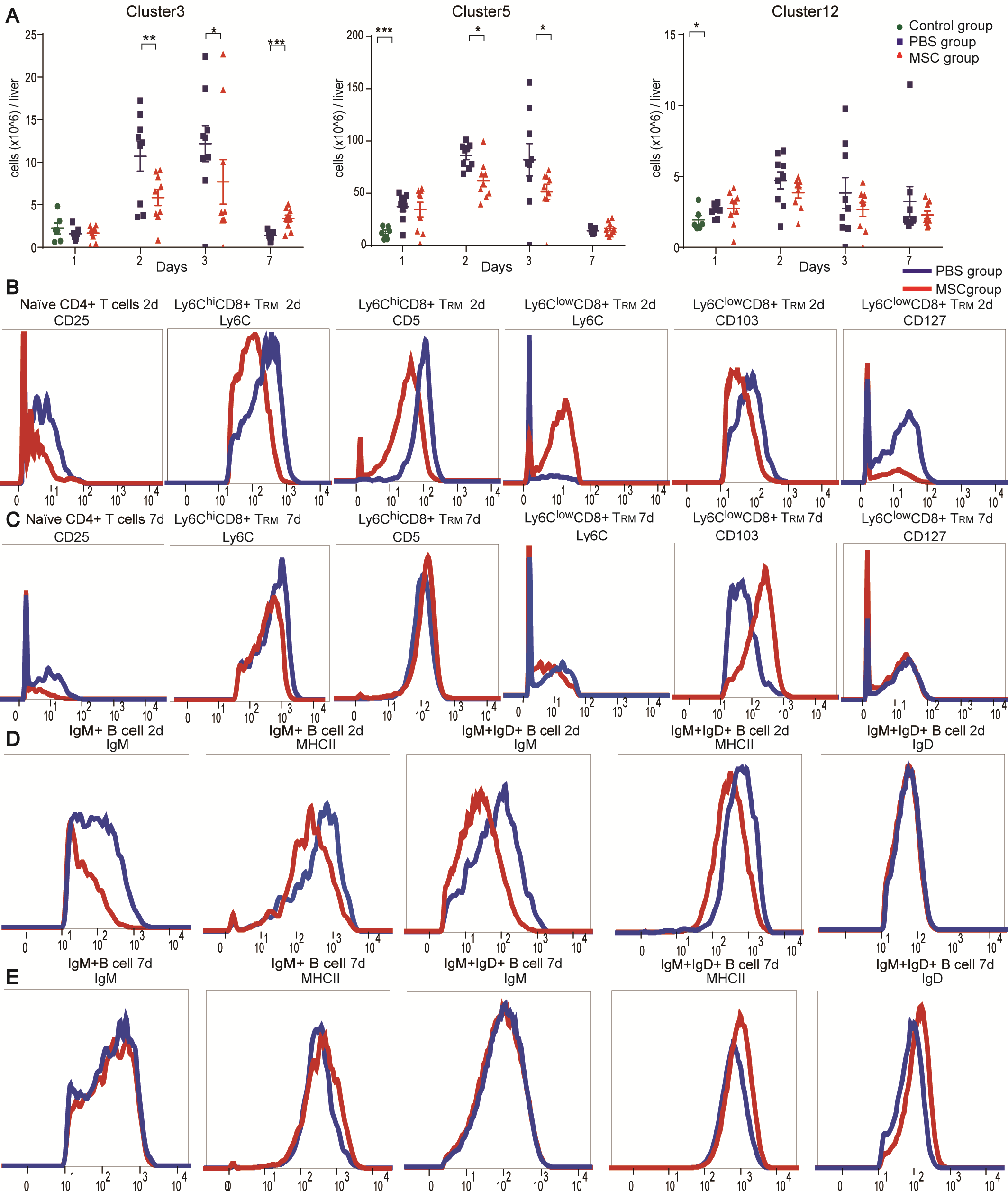
**

**Figure S7. High-dimensional characteristics of MSC-specific alterations in innate immune cell subsets.** **(A):** Representative overlay histograms of the expression of CD62L on cNK cells in the MSC and placebo groups. **(B):** Correlation plot of cNK cells and Ly6C^low^CD8^+^ T_RM_ on day 7. **(C):** Absolute numbers of cluster 9, cluster 15, cluster 17-cluster 21, and cluster 24-cluster 25 over time (n = 8–9, **P <*0.05, ***P <*0.01, and ****P <*0.001 by the paired *t*-test). **(D-E):** Representative overlay histograms of the expression of the selected cell surface markers on moDC and monocyte-derived macrophages in the MSC and placebo groups. **(F):** Correlation plot of moDC with T cells on day 2.


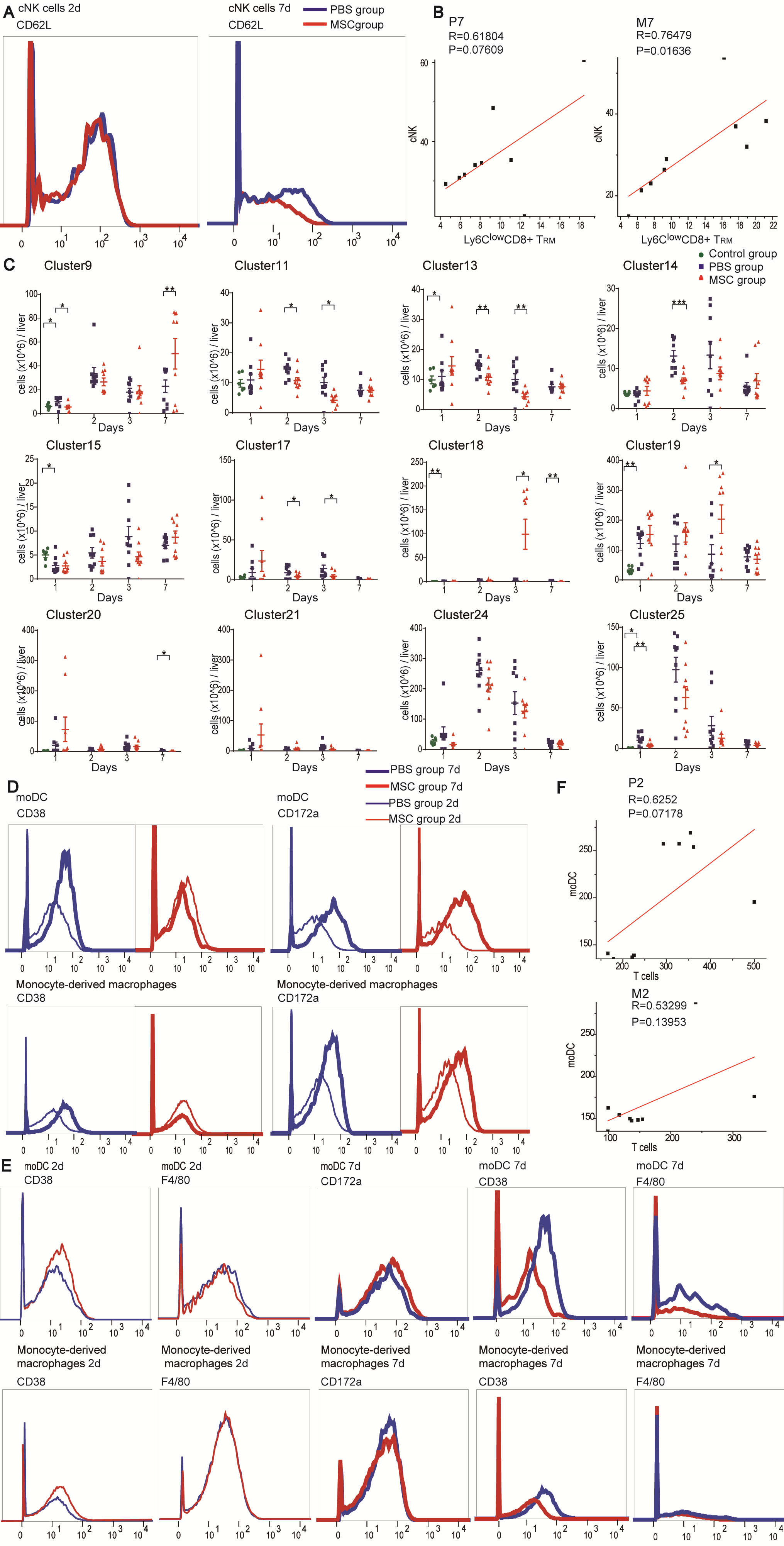


**Figure S8.** **Changes in CD43 after MSC treatment in the injured and recovery phases. (A-B):** Representative overlay histograms of the expression of CD43 on selected cell subsets on day 2 (S8A) and day 7(S8B). **(C):** Bar plots show normalized expression of the CD43 for selected cell subsets (n = 8-9, **P <*0.05, ***P <*0.01, and ****P <*0.001 by the paired *t*-test).


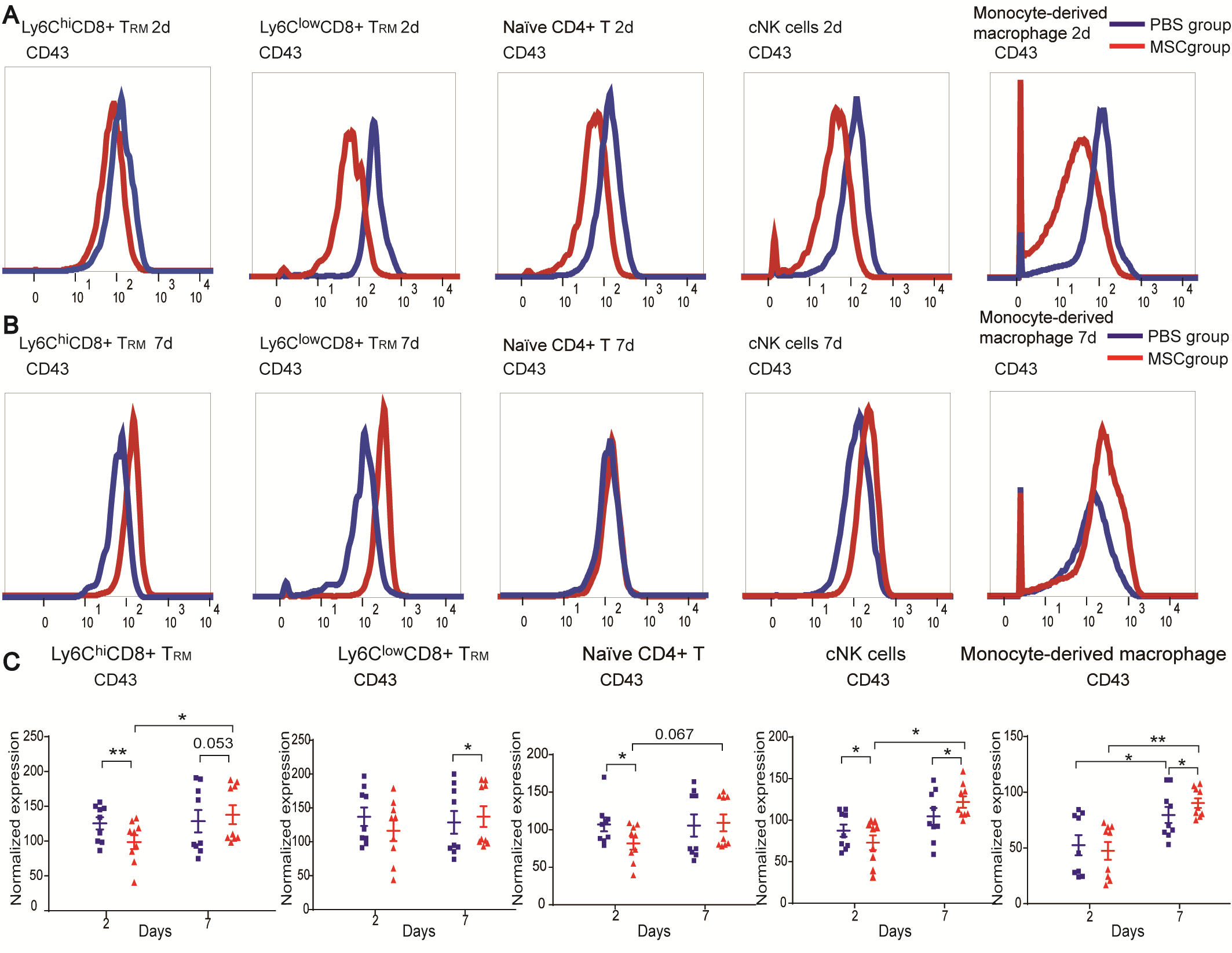

Supplement: Supplementary file 1 — Table S1. List of 43 metal isotope-tagged antibodies for mass cytometry. Table S2. Mean Proportions for all cell subsets of mouse liver immune cells by t-SNE/X-shift in our study. Figure S1. Characteristics of male C57BL/6 mouse MSCs at passage 3. Figure S2. Gating strategy for valid immune cells. Figure. S3. MSCs grafted into CCl4-induced injured livers and biochemical tests. Figure S4. Comparison mass cytometry and flow cytometry analysis of mouse liver four major cell subsets defined by traditional criteria. Figure S5. viSNE map of markers. Figure S6. High-dimensional characteristics of MSC-specific alterations in adaptive immune cell subsets. Figure S7. High-dimensional characteristics of MSC-specific alterations in innate immune cell subsets. Figure S8. Changes in CD43 after MSC treatment in the injured and recovery phases. (DOC 10303 kb) [file 13287_2019_1379_MOESM1_ESM.doc]
